# Phosphoribosyl pyrophosphate synthetases 2 knockdown inhibits prostate cancer progression by suppressing cell cycle and inducing cell apoptosis

**DOI:** 10.7150/jca.37401

**Published:** 2020-01-01

**Authors:** Hui Qiao, Xiao Tan, Dao-jun Lv, Rong-wei Xing, Fang-peng Shu, Chuan-fan Zhong, Chun Li, Ya-guang Zou, Xiang-ming Mao

**Affiliations:** 1 Department of Urology, Nanfang Hospital, Southern Medical University, 510515, Guangzhou, Guangdong Province, China.; 2 Department of Urology, Zhujiang Hospital, Southern Medical University, 510282, Guangzhou, Guangdong Province China.; 3 Nursing Department, Nanfang Hospital, Southern Medical University, 510515, Guangzhou, Guangdong Province, China.; 4 Department of Stomatology, Nanfang Hospital, Southern Medical University, 510515, Guangzhou, Guangdong Province, China.; 5 Department of Urology, the Affiliated Weihai Second Municipal Hospital of Qingdao University, 264200, Weihai, Shandong Province, China.

**Keywords:** apoptosis, Phosphoribosyl pyrophosphate synthetase 2, proliferation, prostate cancer.

## Abstract

Phosphoribosyl pyrophosphate synthetases 2 (PRPS2) protein function as nucleotide synthesis enzyme that plays vital roles in cancer biology. However, the expression profile and function of PRPS2 in prostate cancer (PCa) remain to be identified. Here we investigated the expression of PRPS2 protein in human PCa and paired normal tissues by immunohistochemistry, meanwhile the regulatory effects on cell proliferation, apoptosis and growth of xenograft tumors in nude mice were evaluated in PCa cells with PRPS2 depletion. Moreover, the signaling pathways were also explored by western blot analysis and quantitative polymerase chain reaction assays. We found that PRPS2 was dramatically upregulated in prostate adenocarcinoma tissues in comparison with normal tissues, and that increased PRPS2 was linked intimately to advanced clinical stage and pT status. Functional experiments showed that knockdown of PRPS2 significantly suppressed cell growth both* in vitro* and *in vivo*. In addition, depletion of PRPS2 induced G_1_ phase cell cycle arrest and elevated cell apoptosis. Silencing of PRPS2 resulted in the decreased expression of Bcl‑2 and cyclinD1 and increased levels of Bax, cleavage of caspases‑3, caspases‑9 and PARP. Furthermore, we also detected PRPS2 expression was significantly induced after DHT treatment, which implied the important role of PRPS2 in oncogenesis of PCa. Taken together, our findings elucidated that PRPS2 may be a potential novel candidate for PCa therapy.

## Introduction

Prostate cancer (PCa) is the fifth leading causes of cancer-related death and the most common human carcinoma among men worldwide 1. Androgen deprived therapy (ADT) or platinum-based chemotherapy or other systemic treatment has been proved to partial effectively to alleviate symptoms for advanced prostate adenocarcinoma patients; however, the overall survival benefit is still limited 2. A growing number of evidence highlights that exceptional overexpression of apoptosis resistant proteins, and dysregulation of proliferation-related genes is obsessed in the advance of PCa 3, 4. Whereas, most of the specific elements in the tumorigenesis of PCa remain poorly understood. Hence, it is imperative to find biomarkers that might improve the therapeutic capacity of PCa.

Human phosphoribosyl pyrophosphate synthases isoform 2 (PRPS2) is a key rate-limiting enzyme in the purine biosynthesis pathway, which catalyzes the irreversible conversion of ribose 5-phosphate to phosphoribosyl pyrophosphate (PRPP) with the help of Mg^2+^-ATP 5, 6. Multiple researches declared that PRPS isoforms played quiet different roles between physiology and pathological state, but the mechanisms of functional difference between human PRPS2 and PRPS1 are still uncovered. A recent study showed a special role of PRPS2 in Myc-driven tumorigenesis and identified PRPS2 as a target protein that can manipulate cancer cells both in nucleotide biosynthesis and metabolic rate aspects 7. Interestingly, PRPS2 was reported to contain a consensus pyrimidine-rich translational element (PRTE) motif within its 5' UTR that enables Myc directly increase nucleotide biosynthesis via translational regulation mechanisms to the enhanced protein synthesis of cancer cells 7, 8. Notably, our previous study revealed that the expression of PRPS2 correlated with sertoli-cell only syndrome *via* inhibits cell apoptosis 9. All of these indicated the important role of PRPS2 in the onset and progression of multiple human diseases. However, the detailed function and mechanism of PRPS2 in PCa are still uncertain.

Here, our research was aimed to investigate the precise biological characters of PRPS2 in the development and progression of PCa. Our findings suggested that PRPS2 was frequently upregulated in PCa tissues compared with that in adjacent normal tissues. Additionally, the upregulation of PRPS2 was related to PCa malignant progression. Furthermore, silencing of PRPS2 triggered cell cycle arrest and caspase3-dependent apoptosis. Therefore, PRPS2 may serves as a tumor promoter and a potential new therapeutic target for PCa.

## Materials & Methods

### Clinical specimens

The tissue microarray (TMA) was constructed as described previously 10, using archived pathological formalin-fixed, paraffin-embedded specimens collected from localized PCa patients who underwent radical prostatectomy (RP) or biopsy and confirmed by pathology at the Nanfang Hospital between January 2013 and June 2018. The detailed criteria for the inclusion and exclusion of patients were as follow: Inclusion criteria, 1. Patients diagnosed with PCa before surgery in accordance with biopsy pathological diagnosis; 2. Patients have written the informed consent to collect the specimens. Exclusion criteria, 1. These patients with other malignant disease or a second primary tumor; 2. These PCa patients who received preoperative chemotherapy or radiotherapy before surgery; 3. HIV or syphilis positive patients. All our experimental protocols were authorized by the Ethics Committee of the Southern Medical University Nanfang Hospital. The detailed clinic parameters of enrolled patients, including the age, gender, tumor location, tumor differentiation and tumor node metastasis (TNM) classification, were archived and exhibited in **Table S1**. The pathological diagnoses of enrolled patients were verified by two different pathologists based on the WHO grading system.

### Immunohistochemistry (IHC) analyses

IHC assay was carried on paraffin-embedded tissue sections to determine PRPS2 (1:50, #NBP1-31435, Novus) and Ki-67 (1:200, #ab16667, Abcam) protein expression. The avidin-biotin-peroxidase method was adopted to confirm the location and the relative expression level of the PRPS2. Briefly, tissue slides were deparaffinized with xylene and rehydrated with ethanol, and subjected to boiled retrieval in a sodium citrate buffer for 10 min. Sections were subsequently immersed in 3% H_2_O_2_ for 30 min to quench endogenous tissue peroxidase activity. Subsequently, slides were incubated with primary antibodies overnight at 4 ℃, followed by incubation with the biotin-linked anti-Rabbit secondary antibody (Zhongshan Biotech, China) in combination with the diaminobenzidine (DAB) complex. Slides were counterstained with hematoxylin, then dehydrated and mounted with glass coverslips according to standard protocol. The positive staining intensity of PRPS2 was scored into four categories: 0, negative; 1, weakly positive; 2, intermediately positive; and 3, strongly positive. The percentage of PRPS2 positive cells was scored as four categories: 0, no staining; 1: <25% cells; 2: 25%-75% cells; and 3: >75% cells. Total protein expression score (ranging from 0 to 9) of a sample was obtained by the multiplication of the intensity and percentage scores, as previously described 11. The staining pattern of TMA was scored based on the total protein expression scores as follows: total protein expression score: 0, -; 1-3, +; 4-6, ++; and 6-9, +++. We subsequently divided our cases into two groups using total protein expression scores: cases with total expression score 0-3 (-/+) was regarded as the low expression group, and cases with total expression score 4-9 (++/+++) was regarded as the high expression group. Sections were visualized under a microscope (Olympus, Japan).

### Cell cultures

Human PCa cell lines PC3, DU145, C4-2, LNCaP and the non-malignant immortalized human prostate epithelial cell line RWPE-1 were obtained from Chinese Academy of Sciences cell bank (Shanghai, China). All PCa cell lines were routinely cultured in RPMI-1640 (Gibco, Grand Island, NY, USA) containing 10% fetal bovine serum (Life Technologies, Monza, Italy) and 1% penicillin/streptomycin (Life Technologies). RWPE-1 cells were grown in Keratinocyte Serum Free Medium (KSFM) (Gibco, No. 10724-011) supplemented with 50 mg/mL bovine pituitary extract (Gibco, No. 13028-014), 5 ng/mL epidermal growth factor (EGF) (Gibco, No. 10450-013), and 1% antibiotic-antimycotic solution (Gibco, No. 15240062). Cells were incubated at 37 °C in a humidified atmosphere of 5% CO_2_. LNCaP cells were maintained in Phenol Red-free RPMI 1640 (GIBCO, No. 11835-030) contained with 10% charcoal-dextran-stripped FBS for 72 h before androgen stimulation, then double hydrogen testosterone (DHT) was added to the culture medium.

### RNA extraction and real-time qRT-PCR

Total RNA was isolated with TRIzol reagent (TaKaRa) according to the manufacturer's instructions. Complementary DNA was reverse-transcribed using Prime Script RT reagent Kit (TaKaRa Biomedical Technology, Dalian, China). Quantitative Real-time PCR (qRT-PCR) analysis was conducted using the SYBR^®^ Green PCR Master Mix (Toyobo, Osaka, Japan). The specific primers set for human PRPS2 were: 5'-AGCTCGCATCAGGACCTGT-3' (forward) and 5'- ACGCTTTCACCAATCTCCACG-3' (reverse), and GAPDH primers were 5'-CCAGGTGGTCTCCTCTGACTTC-3' (forward) and 5'-GTGGTCGTTGAGGG CAATG -3' (reverse). All data analyses were managed using the ABI 7500 Fast Analyzer (Life Technologies Corp.). Data were gathered from three biological and technical replicates then normalized to GAPDH expression levels by using the 2^-ΔΔCt^ method.

### Western blotting analysis

Cells were collected in PBS and lysed in RIPA lysis buffer containing proteinase inhibitors (#KGP250, KeyGEN BioTECH, Nanjing, China) according to the manufacturer's protocols. Lysates were vortexed, prepared on ice for 30 min, and centrifuged at 10,000× g for 30 min at 4 °C. The supernatants were transferred into new tubes, and protein concentrations were measured by BCA assay (#KGP906, KeyGEN BioTECH). Equal total of 30 μg cell protein was loaded onto sodium dodecyl sulphate-polyacrylamide electrophoresis gels and electric-transferred onto PVDF membranes (Millipore, Billerica, MA, USA). All membranes were blocked with a Tris/saline solution containing 5% non-fat milk and 0.1% Tween-20 for 1 h and probed with specific primary antibody: PRPS2 (#NBP1-31435, rabbit, Novus), Caspase-3 (#9665, rabbit, Cell Signaling Technology), Cleaved Caspase-3 (#9664, Cell Signaling Technology), PARP (#9542, Cell Signaling Technology), Cleaved PARP (#5625, Cell Signaling Technology), Cleaved Caspase-9 (#52873, Cell Signaling Technology), Caspase-9 Mouse mAb (#9508, Cell Signaling Technology), cyclin D1(#2978, Cell Signaling Technology), p27 Kip1(#3686, Cell Signaling Technology), CDK4 (#12790, Cell Signaling Technology), anti-p53 (ab131442, Abcam), β-actin Mouse mAb (#60008-1-Ig, Proteintech) and α-Tubulin rabbit mAb (#11224-1-AP, Proteintech) antibodies. After extensive washing, membranes were incubated with secondary conjugated to horseradish peroxidase (1:10,000; Cell Signaling) for 1 h at room temperature. Proteins were visualized and detected using ECL kit (Thermo; Rockford, IL).

### siRNA transfection and establishment of stably transfected cell line

Three siRNAs targeting PRPS2 (si-PRPS2-1, 2, 3) and negative control siRNA (NC) with no definite target were purchased from by RiboBio Company (Guangzhou, China). The sequences of siRNA oligonucleotides are shown as below, si-h-PRPS2_001: CTGCAAGATTGCGTCATCA; si-h-PRPS2_002: CCACCAAAG TGTATGCTAT; si-h-PRPS2_003: GAAACACTGCACCAAGATT. The three siRNAs and NC were transfected using Lipofectamine^TM^ 3000 (Invitrogen, Carlsbad, CA, USA) as described in the manufacturer's instructions. After 48 h of transfection, total RNA or protein was extracted for qRT-PCR or immunoblot analysis as described above. Stable knockdown of PRPS2 in PC-3 cells were achieved by lentivirus vector (GeneChem BioMedical Biotechnology, Shanghai, China). Briefly, cells at 30% to 40% confluence were infected by PRPS2 shRNA (CCACCAAAGTGTATGCTAT) lentivirus and empty lentivirus vector, respectively. Thereafter, stable cell lines were selected by puromycin (5 µg/mL) for one week. Then Western blot was conducted to confirm the efficiency of depletion of PRPS2 with short hairpin RNAs.

### Cell proliferation assay

Cell growth was evaluated using the CCK-8 assay (CK04, Dojindo, Japan). PC-3 and DU145 cells were plated in 96-well plates at a density of 2 × 10^3^ per well at 24 h after transfection. Then CCK-8 reagent (1:100) was added into wells at different time points and incubated for 2 h. The absorbance values at 450 nm were determined using a microplate reader (Dynex Technologies, Inc., Chantilly, VA).

### Colony formation Assay

Cells were seeded onto 6-well plates in triplicate at a density of 1 × 10^3^ cells/well in flat-bottomed six-well plates, and then cultured in complete medium for two weeks. The colonies were fixed in 4% paraformaldehyde before stained with Wright Giemsa stain (Baso Diagnostics Inc. Zhu Hai, China) for 10 min for colony counting.

### 5-ethynyl-20-deoxyuridine assay (EdU) incorporation Assay

Each group of PCa cells were seeded onto 24-well plates with a density of 10^5^/well. Then the cells were incubated in the respective media containing 50 μM EdU (RiboBio, Guangzhou, China) for 2 h. Cell proliferation was conducted utilizing a Cell-Light™ EdU DNA Cell Proliferation Kit (RiboBio, Guangzhou, China) according to the manufacturer's protocols. Nuclear DNA was stained with Hoechst33342 stain (200 μL/well) for 30 min. Images were visualized with an inverted fluorescence microscope at 100× (Olympus, Tokyo, Japan). The percentage of EdU positive cells (with red fluorescence) to Hoechst nuclear staining cells (with blue fluorescence) was analyzed using ImageJ software to determine the cell proliferation activity.

### *In vitro* cell apoptosis analysis

Flow cytometry analysis for apoptosis was performed by utilizing Annexin V-FITC Apoptosis Detection Kit (#KGA108-1, KeyGEN BioTECH) after 48 h transfection according to the kit's protocol. Cell apoptosis were validated by FACS Caliber FCM (Becton Dickinson Biosciences, San Jose, CA) and histograms generated by using the Flow Jo software package (Tree Star, Inc.). All experiments were analyzed in triplicates.

### Cell cycle analysis

Cells were harvested by tyrisin and washed slightly three times in PBS, then resuspended and cool-fixed at 4 °C in 70% ethanol solution overnight. After centrifuge to discard the ethanol we washed cells in PBS three times, then cells were immersed in 10 μg/mL RNase A (Sigma. USA) solution for 30 min at room temperature. Subsequently, we stained the cells with propidium iodide (PI, 50 μg/mL) in the dark place. Finally, DNA content of each sample was measured by flow cytometry.

### Tumor xenografts in Nude mouse

For the tumor xenografts experiments, total of 5 × 10^6^ stable transfected with PRPS2 shRNA (PC-3/sh-PRPS2) or negative control (PC-3/sh-Ctrl) PC-3 cells in 100 µL of PBS were injected subcutaneously into the right flanks of nude male mice (n = 8, per group). Xenograft size was measured using caliper every 3 days, and volumes were evaluated using the formula: A × B^2^ × 0.5 (A represent length and B represent width). At 26 days post injection, mice were euthanized, the tumor specimens were carefully excised, photographed and tissues were preserved for further histologic evaluation (paraffin section) including hematoxylin and eosin (H&E), IHC and terminal deoxynucleotidyl transferase dUTP nick end labeling (TUNEL). All BALB/c nude mouse experiments were approved by the Institutional Animal Care and Use Committee of Southern Medical University.

### TUNEL assays

All xenograft tumor tissues were fixed with formaldehyde solution for 24 h, dehydrated and embedded in paraffin. Tunnel kit (#KGA7051, Nanjing KeyGen Biotech Co., Ltd.) was applied to identify apoptosis cells of tumors according to the manufacture's instruction. Briefly, three micrometer-thick deparaffinized sections were deparaffinized, rehydrated and quenched with 1% proteinase K (20 mg/mL) at 37 °C for 30 min. Subsequently, specimens were washed with PBS and inculcated with TUNEL mixture (45 μL Equilibration Buffer, 1.0 μL biotin-11-dUTP, 4.0 μL TdT Enzyme, and 50 μL reaction buffer) in the dark for 1 h at 37 °C. After washing with PBS, the specimens were stained with DAPI. Apoptotic cells were stained as red and photographed using fluorescence microscopy at 570 nm.

### Statistical analysis

Statistical analysis was performed with SPSS 20.0 software (SPSS, Chicago, USA). The data are presented as means ± standard deviation (SD) of at least three repeats. The significance of mean values were analyzed using for continuous variables ANOVA or Student's t-test. *Pearson's* chi-square test was applied to analyze the clinical variables. *P* value < 0.05 was considered as statistically significant. (^*^P < 0.05, ^**^P < 0.01, and ^***^P < 0.001).

## Results

### PRPS2 expression levels in PCa tissues and cells

**Figure 1:**
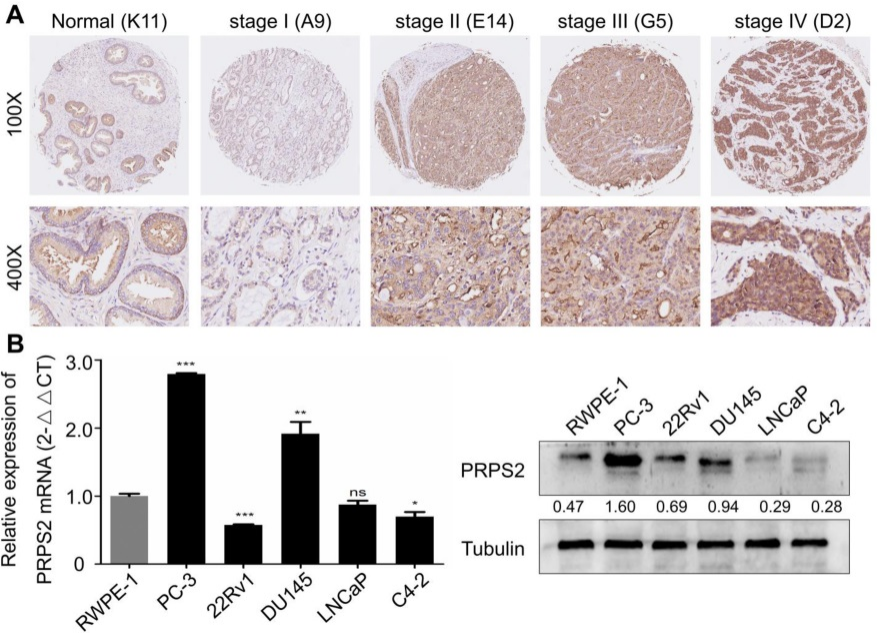
** PRPS2 expression in prostate cancer tissues and prostate cancer cell lines. (A)** Representative immunohistochemistry images of PRPS2 protein expression from the whole TMA with low intensity (b1) in normal prostate tissue (K11, scale bar = 0.2 mm), with low intensity (b4) in prostate cancer tissue (A9, scale bar = 0.2 mm), with intermediate intensity (b3) in prostate cancer tissue (G5, scale bar = 0.2 mm), and with high intensity (b2) in prostate cancer tissue (D2, scale bar = 0.2 mm). The percentage of PRPS2 positive cells in D2 is 90%, in G5 is 70%, and in A9 is 15%. Therefore, the percentage score of the case in D7 is 3 and its total protein expression score is 3 × 3 = 9. The percentage score of the case in H7 is 2 and its total protein expression score is 2 × 2 = 4. The percentage score of the case in E4 is 1 and its total protein expression score is 1 × 1 = 1. **(B)** Western blotting and real-time qPCR analysis of PRPS2 protein and mRNA expression in prostate cancer cell lines and normal prostate epithelial cell lines (RWPE-1). (^*^P < 0.05; ^**^P < 0.01; ^***^P < 0.001). PRPS2: Phosphoribosyl pyrophosphate synthetase 2; TMA: tissue microarray.

To detect PRPS2 expression in PCa, we performed IHC analysis of a human prostate tissues microarray. Based on PRPS2 staining levels (**Fig. 1A‑B**), all prostate tissues were divided into two groups: a low expression group (- and +) and a high expression group (++/+++). Results demonstrated that PRPS2 was frequently up-regulated in PCa tissue samples (60/78, 76.9%). Whereas the PRPS2 positive rate in the nonneoplastic (normal or adjacent) tissues was significantly lower (7/16, 43.8%). Chi‑Square analysis showed that PRPS2 expression levels were higher in PCa tissues than in nonneoplastic (normal or adjacent) tissues (Table 1, χ^2^ = 5.608, *P =* 0.018). Then, we further confirmed the expression of PRPS2 mRNA and protein in five PCa cell lines (PC‑3, 22Rv1, DU145, LNCaP, and C4-2) and a noncancerous prostatic epithelial cell (RWPE-1). Two out of five PCa cells had increased PRPS2 protein and mRNA expression level compared with RWPE-1 cells (Fig. 1C). We then assessed the relationship between PRPS2 and clinicopathologic variables. Chi‑Square analysis demonstrated PRPS2 protein expression level was positively correlated with clinical stage (Table 1, χ^2^ = 5.114, *P* = 0.024), and pT status (Table 1, χ^2^ = 5.966, *P* = 0.015), but was not linked with other clinicopathological features (Table 1). In a word, these findings strongly suggested that PRPS2 expression was associated with PCa invasion and migration. Taken together, these results indicate that PRPS2 was up-regulated in PCa and may be involved in the tumorigenesis of PCa.

### Silencing of PRPS2 represses PCa cell proliferation *in vitro*

**Figure 2:**
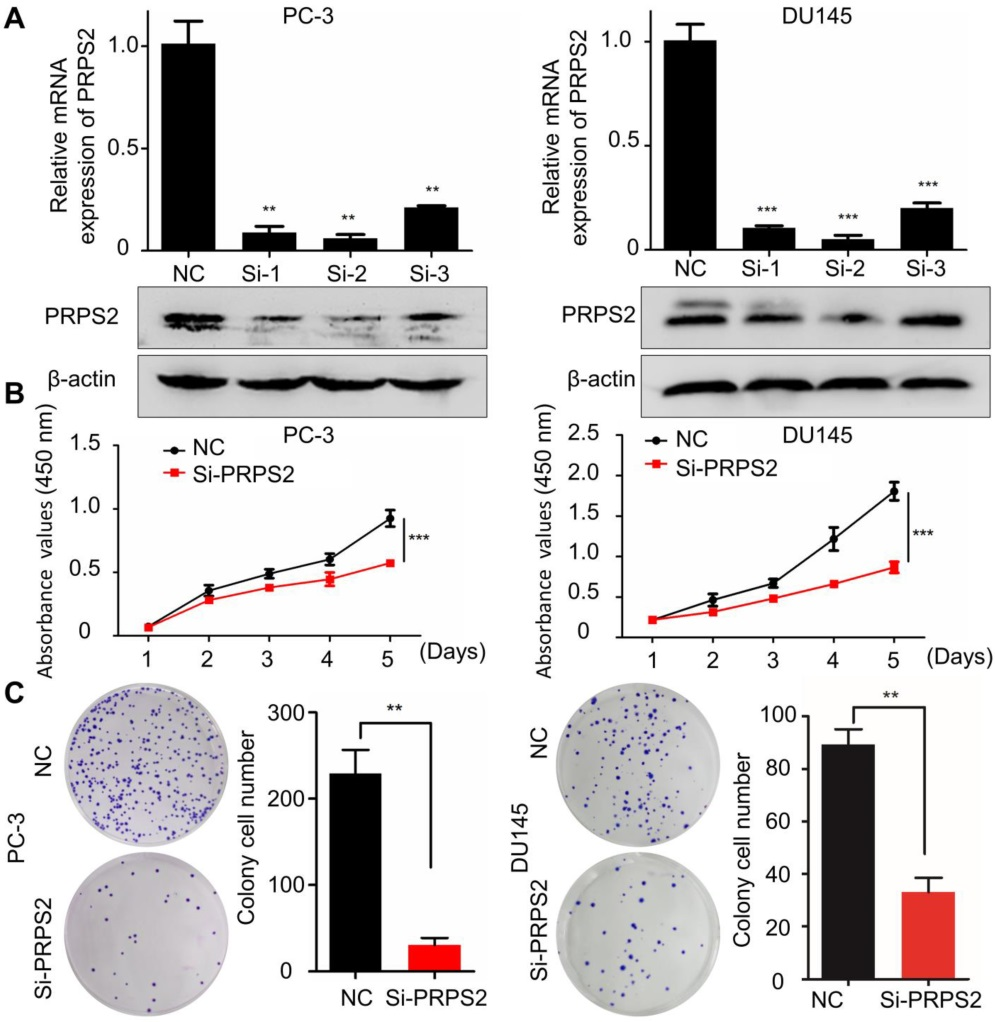
** Knockdown of PRPS2 decreased prostate cancer cell growth. (A)** The effects of PRPS2 knockdown confirmed by qRT-PCR (top) and Western blotting (bottom). **(B)** In the CCK‑8 assay, cell viability was decreased in si‑PRPS2 compared with NC. **(C)** In the plate colony formation assay, colony formation was obviously decreased in si-PRPS2 cells transfected. ^***^P < 0.001. NC: negative controls.

To determine the biological function of PRPS2 in PCa, siRNA targeting PRPS2 (si‑PRPS2) was transfected into PC‑3 and DU145 cells to inhibit endogenous PRPS2 expression. qRT-PCR and Western blot confirmed that PRPS2 was markedly decreased in PC‑3 and DU145 cells transfected with si‑PRPS2 compared with negative control (NC) cells (**Fig. 2A**). Then we performed CCK‑8 and colony formation assays to detect the effect of PRPS2 on cell proliferation. Results demonstrated that PRPS2 knockdown markedly suppressed cell growth and capacity to form colonies of PC‑3 and DU145 cells compared with NC cancer cells (**Fig. 2B-C,** *P* < 0.001).

### PRPS2 knockdown induced cell cycle arrest and apoptosis in PCa cells

**Figure 3:**
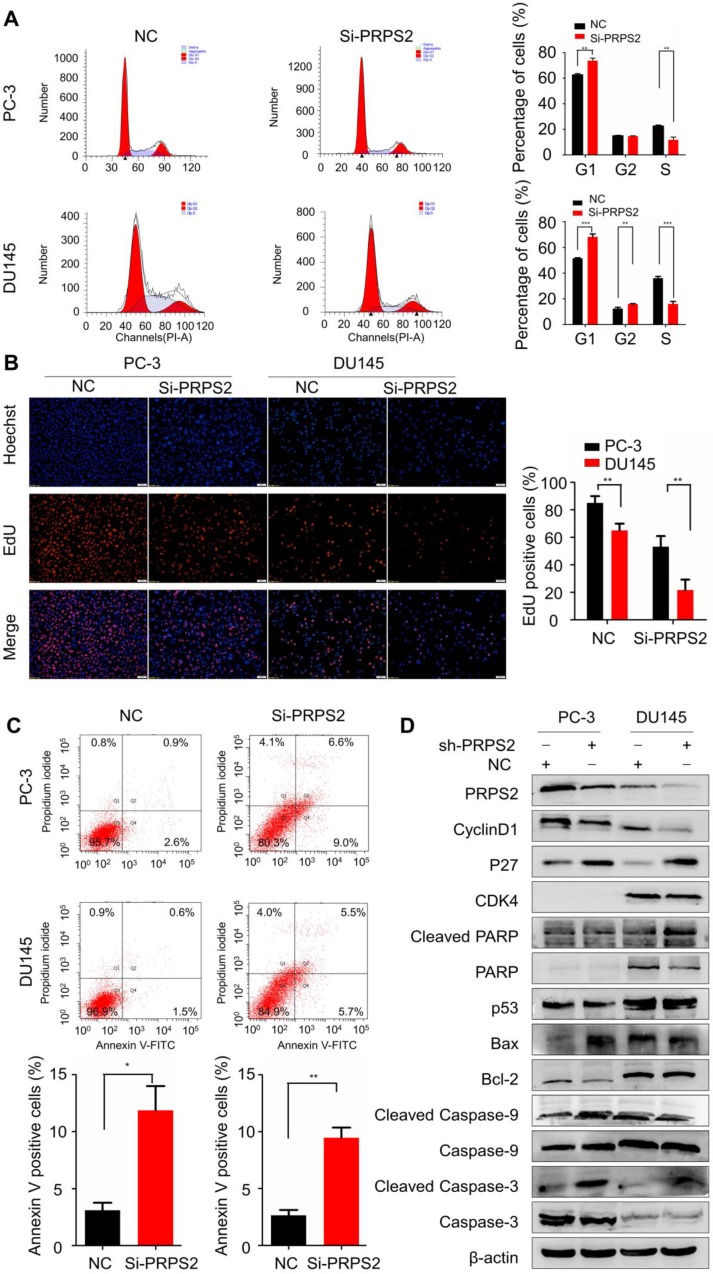
** Knockdown of PRPS2 induced prostate cancer cell apoptosis and cell cycle arrest. (A)** PRPS2 knockdown induced PCa cell cycle arrested in G_0_/G_1_ phase. **(B)** Representative micrographs (left) and quantification (right) of EdU incorporation assay. ^**^P < 0.01, ^***^P < 0.001. **(C)** PRPS2 knockdown increased apoptosis rate. ^*^P < 0.05. **(D)** Western blot showed the protein levels of cell apoptosis-related proteins (cleavage of caspase-3, caspase-9, PARP, Bcl-2, Bax and p53) and cell cycle-related proteins (p27, CDK4 and cyclin D1) after down-regulated expression of PRPS2 in PC-3 (left) and DU145(right) cells. β-actin was served as loading control. NC, negative control.

Then we performed flow cytometry to validate whether the PRPS2-depletion suppresses progression through cell cycle. The inhibition of PRPS2 caused a significant increasing of cellular DNA content in G_1_ phase (Fig. 3A, *P_PC-3_*=0.0012, *P_DU145_*<0.001). Similarly, EdU incorporation assays revealed that knockdown of PRPS2 markedly inhibited cell proliferation as compared to controls (Fig. 3B, *P_PC-3_* = 0.0039, *P_DU145_* = 0.0012). These results of *in vitro* experiments indicated that knockdown PRPS2 inhibited the proliferation of PCa cells.

Next, we used flow cytometry to identify the influence of PRPS2 expression on apoptosis. Results showed that apoptosis rates were markedly increased among cells transfected with si‑PRPS2 compared with NC cells. Student's *t*‑test analysis revealed that the mean total number of apoptosis cells increased from 3.10 ± 1.15 to 11.87 ± 3.70 and from 2.63 ± 0.84 to 9.47 ± 1.55 in response to PRPS2 knockdown in PC‑3 cells (*P* = 0.0173) and DU145 cells (*P* = 0.0026), respectively (Fig. 3C).

To further reveal the potential mechanism underlying this cell cycle arrest and apoptosis promotion by PRPS2 knockdown, we detected the expression of apoptosis‑related markers (Bcl‑2, Bax, P53, PARP1, caspase‑3, caspase‑9, cleaved PARP1, cleaved caspase‑3 and cleaved caspase‑9) and cell cycle related proteins (cyclin D1, P27, and CDK4) by Western blotting. As showed in **Figure 3D**, the apoptosis-promoting proteins Bax, cleaved PARP1, cleaved caspase‑3 and cleaved caspase‑9 were substantially increased in response to PRPS2 knockdown compared with NC cancer cells. Conversely, the apoptosis-inhibiting proteins expression of Bcl‑2 decreased in si‑PRPS2‑transfected PCa cells. With regards to cell cycle related proteins silencing of PRPS2 significantly increased cyclin-dependent kinase inhibitor P27, whereas cyclin D1 which functions as a mitogenic sensor and allosteric activator of CDK4/6 decreased remarkably. Therefore, these results indicated that PRPS2 depletion activated intrinsic apoptosis by activating PARP/Bcl2/Caspase pathways and induced cell cycle G_1_ arrest at least partly attributed to cyclin-dependent kinase.

### Knockdown of PRPS2 inhibited xenograft tumor growth *in vivo*

**Figure 4:**
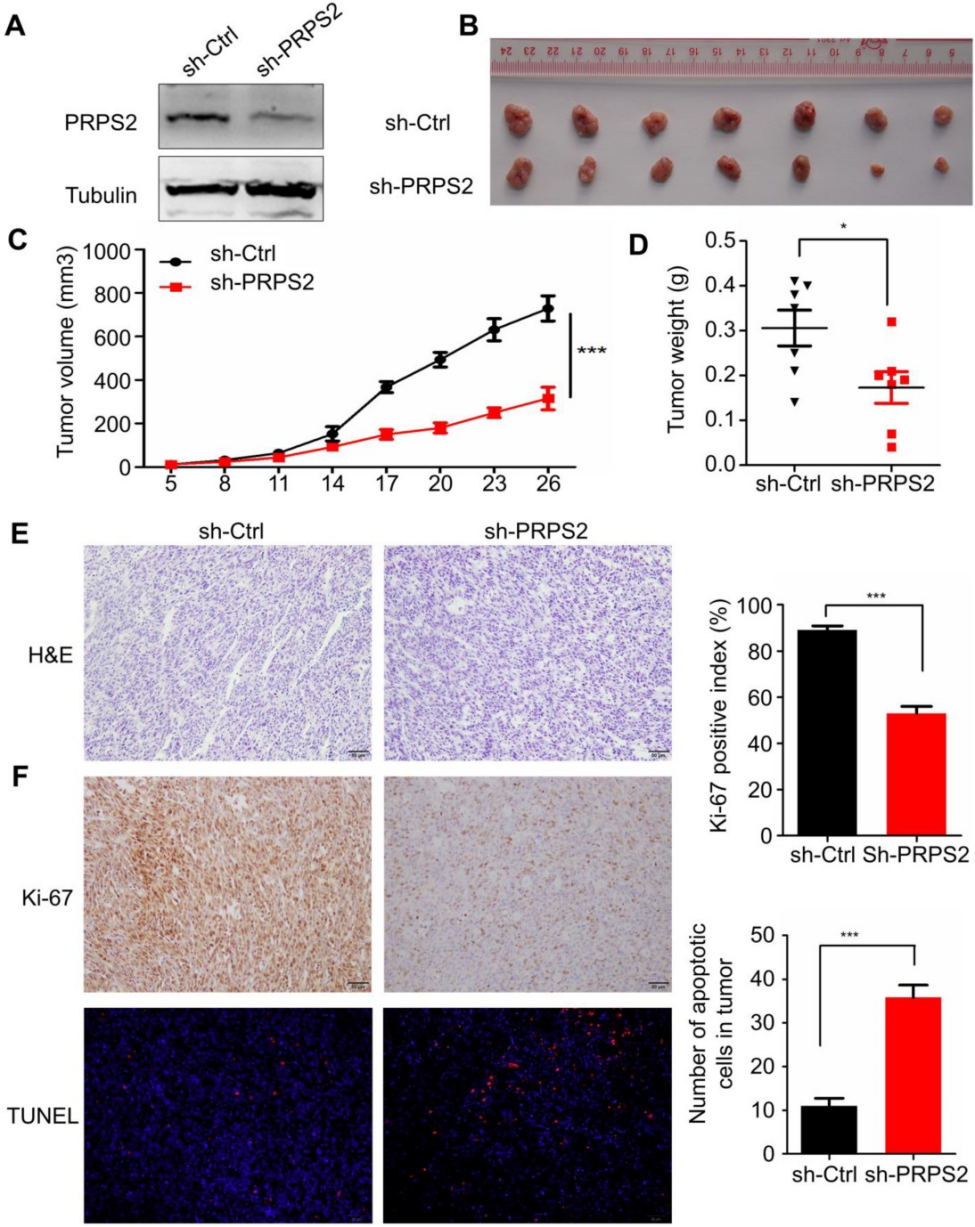
** Knockdown of PRPS2 inhibits xenograft tumor growth *in vivo*. (A)** PRPS2 shRNA effectively blocked its protein expression in PC-3 cells. Cells were stably transfected with scramble (sh-Ctrl) or PRPS2 shRNA (sh-PRPS2). PRPS2 protein levels were normalized to α-Tubulin. **(B)** Gross observation of xenograft tumor size in NOD/SCID mice. **(C and D)** Silencing of PRPS2 inhibited the tumor growth, including tumor volume (*P* < 0.001) and weight (*P* = 0.028, n = 6). **(E)** H&E‑stained paraffin‑embedded sections obtained from xenografts of PC-3 cells. **(F)** Top: Immunohistochemical analysis of Ki-67 in the xenografts; Bottom: The apoptosis in tumor tissues was evaluated by TUNEL assay (×200). Graphical illustrated the quantification of Ki-67 and TUNEL positive cells percentage. ^***^P < 0.001.

To evaluate the impact of PRPS2 knockdown on tumor growth *in vivo*, we established a subcutaneous xenograft tumor model in athymic nude mice by injecting PC‑3 cells infected with shRNA targeting PRPS2 or NC PC‑3 cells. Results from Western blotting showed an obvious reduction of PRPS2 expression in PC‑3 cells transfected with sh‑PRPS2 compared with NC cells (Fig. 4A). As expected, cells with PRPS2 knocked down formed slower‑growing xenografts compared with NC cells (Fig. 4B‑C, *P* < 0.001). Correspondingly, tumor weight in PRPS2 depletion group was lighter than the control group (Fig. 4D, *P* = 0.028). H&E staining revealed histopathological features of the xenograft tumor tissues (Fig. 4E). Moreover, IHC results of the xenograft tumor tissues demonstrated that the expression of Ki-67 proliferation antigen in xenografts of sh-PRPS2 cells was significantly decreased (Fig. 4F, upper panel, *P* < 0.001). TUNEL staining also showed an apoptosis enhancement of tumor cells after PRPS2 knockdown (Fig. 4F, lower panel,* P* < 0.001). All these results further indicated that PRPS2 knockdown suppressed cell growth at least partially mediated by the elevated intrinsic apoptosis of PCa cells.

### Androgen treatment could induce PRPS2 expression

**Figure 5:**
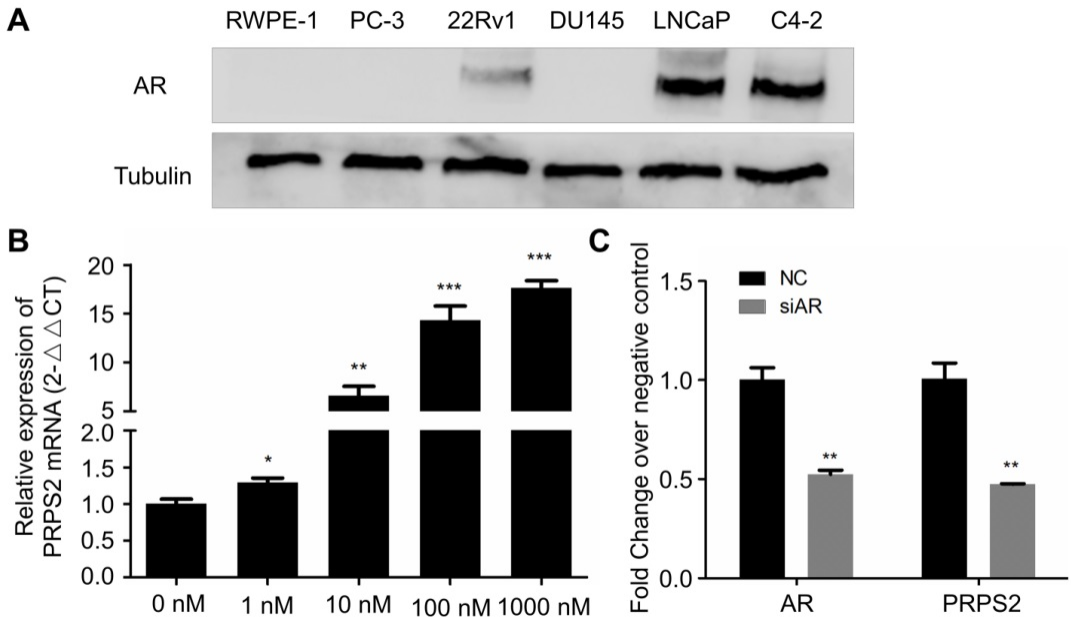
** PRPS2 was an androgen-responsive gene. (A)** Western blotting of AR protein expression in prostate cancer cell lines and normal prostate epithelial cell lines (RWPE-1). **(B)** qRT-PCR analysis of PRPS2 expression with androgen-starved for 72 h and treated with DHT 48 h in dose series of 0 nM, 1 nM, 10 nM, 100 nM, 1000 nM in LNCaP. The expression of PRPS2 after DHT stimulation was normalized to the expression without DHT stimulation. **(C)** The efficiency of siAR on the expression of AR was confirmed by qRT-PCR and the expression of PRPS2 was tested after transfection of siAR compared with NC in LNCaP cells. Data are presented as the mean ± SD (n = 3). (^*^P < 0.05; ^**^P < 0.01; ^***^P < 0.001).

Emerging evidence had proved that androgen receptor (AR) played the most vital roles in PCa development through regulating androgen-responsive genes 12. Interestingly, our previous study revealed that PRPS2 expression involved in sertoli-cell only syndrome (SCOS) and inhibits the apoptosis of sertoli cells via the p53/Bcl-2/caspases signaling pathway 9. Besides, testis AR is exclusively upregulated in SCOS than in normal testis 13. Thus, we hypothesized PRPS2 may be also an androgen regulated gene. To confirm this, we employed dose-dependent DHT treatment to detect whether the expression of PRPS2 is androgen-related in LNCaP cells. Firstly, we confirmed the expression of AR was decisive in 22Rv1, LNCaP and C4-2, whereas PC-3, DU145 and RWPE-1 cells demonstrated negative expression of AR (Fig. 5A). Then qRT-PCR analysis of PRPS2 expression with androgen-starved for 72 h and treated with DHT for 48h in LNCaP cells. Results demonstrated that PRPS2 expression increased 17.6fold (Fig. 5B, *P* < 0.001) after high dose of DHT (1000 nM) stimulation compared with control. Moreover, we also detected that PRPS2 expression was reduced by 53% over negative control (NC) following si-AR transfection (Fig. 5C, *P =* 0.0026). The results implied that PRPS2 may be an androgen-responsive gene.

## Discussion

In this study, we found that PRPS2 was upregulated in PCa tissues compared with normal prostate tissues. Moreover, patients with higher clinical stage and pT status exhibited increased PRPS2 expression. Then we performed loss of function experiments *in vitro* and revealed that silencing of PRPS2 could remarkably suppress the cell proliferation, which might attribute to cell cycle arrest and PRAP-caspase dependent cancer cell apoptosis. In addition, knockdown of PRPS2 drastically inhibited the xenograft tumor growth *in vivo.* These results suggested that PRPS2 may involve in progression and aggressiveness in PCa.

The ability to modify metabolic output to fulfill the biosynthetic and bioenergetic demands of cell growth and proliferation is a defining feature of cancer cells 14, 15. The Myc oncogene regulated several cellular responses including promoting protein synthesis and nucleotide synthesis, imperative for sustaining cancer cell proliferation. PRPS2 is a key rate-limiting enzyme within the urine synthesis pathway which has been reported to be translational upregulated by Myc-driven hyperactivation of protein synthesis 7. There are three isoforms of homo-sapiens PRPS family on record, the amino acid homology of PRPS1 and PRPS2 is approximately 95% 5, PRPS can regulate cancer metabolism and promotes nucleotide synthesis under tumor energy stress 16. As an important rate-limiting enzyme of the pentose-phosphate pathway, PRPS2 boosts increased nucleotide biosynthesis *via* specialized cis-regulatory elements with the PRSP 5`UTR 7. In addition, PRPS2 was confirmed to catalyze the reaction of converse ribose 5-phosphate into 1-5-phosphoribosyl pyrophosphate (PRPP) which is irreversible 17, 18. Recently studies demonstrated that PRPS2 was involved in cancer cell proliferation and metastasis of neuroblastoma malignancy, which was regulated by both MYCN and activating enhancer binding protein-4 (TFAP4) 19. Moreover, pervious study has suggested that PRPS2 may act as an oncogene in PCa on account of PRPP is a key precursor of purine synthesis 20. Meanwhile, lowering serum uric acid levels in PCa was regarded as therapeutically beneficial 21. However, the biological function of PRPS2 in PCa remains largely unknown. Thus, we speculated the occurrence and progress of PCa may be closely related to the unusually exuberant nucleic acid metabolism that was regulated by PRPS2, which provided new evidence for us to learn the abnormal metabolism of PCa cells. As expected, we identified that PRPS2 may be act as an oncogene role to facilitate the growth of PCa cells.

It has become a truism that sustaining proliferation is an important hallmark of cancer 22. c-MYC has been founded an elevated expression in PCa and multiple studies shown c-MYC can induce tumor cells proliferation and regulate cell cycle though MYC-responsive genes 7, 23-25. Cell cycle genes expression level has been founded that associate with the timing of PCa metastasis, as an independent factor 26. Thus, cell cycle related genes or proteins have significant prognostic value in patients with PCa. Furthermore, it has been reported that glucose deprivation can drive brain tumorigenesis *via* c-MYC/AMPK direct phosphorylation of PRPS1 S180 and PRPS2 S183 16, indicated PRPS2 is a c-MYC pathway downstream gene. Myc-overexpressing cells lacking PRPS2 have a decreased ability to increase protein synthesis, possibly because of the reduced nucleotide production required to synthesize ribosomes. Thus, in this study, our data discovered that PRPS2 knockdown significantly upregulated cyclin-dependent kinase inhibitor p27 but downregulated G_1_/S-specific protein cyclin D1 both in PC3 and DU145 cells, indicated that PRPS2 may be a potential druggable target for c-MYC responsive cell cycle checkpoint pathway in PCa.

Resisting apoptosis is common in all kinds of tumors. Dysregulation of apoptosis is also regarded as a hallmark of PCa 27-29. Hence, restore the normal apoptotic cell death in PCa is a critical treatment strategy 30-32, such as androgen deprivation therapy 33. However, after current ADT therapy mostly advanced PCa development to castration-resistant prostate cancer (CRPC), force physicians to find different strategies according to novel AR-related mechanisms 34. Purinergic receptor has been reported to involve in mitochondrial apoptosis via either androgen dependent or independent PCa cells 35. Our study demonstrated that PRPS2 enhanced the ability of apoptosis resistant both in PC-3 and DU145. Further, we founded that the protein expression levels of Bax, cleaved PARP and cleaved caspase-3 in PCa cells were significantly increased in PRPS2 knockdown than controlled groups. Indicated that PRPS2 boosts PCa development *via* inhibited Bax-caspase9/3-PARP dependent apoptosis, which has not been reported yet.

Interestingly, our previous study has revealed that PRPS2 inhibits the apoptosis of TM4 Sertoli cells via the caspases depended signal pathway 9. Besides, testis AR is exclusively overexpression in Sertoli cells only syndrome than in normal testes 13. Recent literatures reported a gene aberrations analysis in PCa before and after proceeding ADT treatment suggested MYC may involvement in CRPC development 36. These studies imply a relationship between AR and MYC related genes. Here, our results demonstrated that DHT stimulation enhanced PRPS2 expression in a dose-dependent manner, which further indicated that PRPS2 may be a testosterone-sensitive enzyme and partly contributed to the occurrence and progress of PCa. However, whether there is a regulatory relationship between abnormal PRPS2 and androgen receptors still needs further studies.

## Conclusion

In summary our study has presented that high PRPS2 expression correlated with the aggressive clinical feature of PCa. Moreover, PRPS2 knockdown efficiently inhibited PCa cell proliferation partly owes to induce cell cycle arrest and facilitate caspase-dependent apoptosis. These results indicated that PRPS2 may play a crucial role in the progression and aggressiveness of PCa, interfering with PRPS2 signaling may develop therapeutic value against PCa with high expression of PRPS2.

## Supplementary Material

Supplementary figure 1.

Supplementary table 1.

## Figures and Tables

**Table 1:** The correlation between PRPS2 expression and clinicopathological characteristics was analyzed in prostate cancer by IHC (n = 78).

Variables	Total N	PRPS2		χ^2^	*p* value^b^
		High expression (++/+++, n, %)	Low expression (-/+, n, %)		
Type					
Normal/BPH	16	7 (43.8)	9 (56.3)	**5.608**	**0.018**
PCa	78	60 (76.9)	18 (23.1)		
Age(years)					
≤ 66^a^	39	26 (66.77)	13 (33.3)	0.692	0.406
> 66	55	41 (74.5)	14 (25.5)		
Clinical stage					
I- II	42	28 (66.7)	14 (33.3)	**5.114**	**0.024**
III- IV	35	31 (88.6)	4 (11.4)		
Primary tumor					
T1-T2	41	27 (65.0)	14 (34.1)	**5.966**	**0.015**
T3-T4	37	33 (89.2)	4 (10.8)		
Gleason Score					
≤7	31	23 (74.2)	8 (25.8)	0.265	0.607
≥8	48	38 (79.2)	10 (20.8)		

**Notes:** a, mean age. b, *p* value is from χ^2^-test -test. ‑/+, total expression score 0‑3; ++/+++, total expression score 4‑9. **Abbreviations:** PCa, prostate cancer.

## Abbreviations

5'-UTR: 5'- Untranslated Regions
ADT: Androgen deprivation therapy
AR: Androgen receptor
CRPC: Castration-resistant prostate cancer
DAB: Diaminobenzidine
DHT: Double hydrogen testosterone
EdU: 5-ethynyl-20-deoxyuridine assay
EGF: Epidermal growth factor
IHC: Immunohistochemistry
KSFM: Keratinocyte serum free medium
PI: Propidium iodide
PRPP: Phosphoribosyl pyrophosphate
PRPS: Phosphoribosyl pyrophosphate synthetases
PRPS2: Phosphoribosyl pyrophosphate synthetases 2
PRTE: Pyrimidine-rich translational element
TMA: Tissue microarray
TNM: Tumor node metastasis
