# Supplementary material for: Phosphoribosyl pyrophosphate synthetases 2 knockdown inhibits prostate cancer progression by suppressing cell cycle and inducing cell apoptosis
Source: J Cancer. 2020 Jan 1;11(5):1027–37. doi: 10.7150/jca.37401 (PMC6959080; doi:10.7150/jca.37401)
Supplement: Supplementary file 1 — Supplementary figure 1. [file jcav11p1027s1.pdf]

Phosphoribosyl pyrophosphate synthetases 2 knockdown inhibits prostate cancer progression by suppressing cell cycle and inducing cell apoptosis

Running title: Role of PRPS2 in prostate cancer

Hui Qiao[3#], Xiao Tan[2#], Dao-jun Lv[1,2#], Rong-wei Xing[5], Fang-peng Shu[2], Chuan-fan Zhong[2], Chun Li[3], Ya-guang Zou[4*], Xiang-ming Mao[1,2*]

[1] *Department of Urology, Nanfang Hospital, Southern Medical University, 510515, Guangzhou, Guangdong Province, China.*

[2] *Department of Urology, Zhujiang Hospital, Southern Medical University, 510282, Guangzhou, Guangdong Province China.*

[3] *Nursing Department, Nanfang Hospital, Southern Medical University, 510515, Guangzhou, Guangdong Province, China.*

[4] *Department of Stomatology, Nanfang Hospital, Southern Medical University, 510515, Guangzhou, Guangdong Province, China.*

[5] *Department of Urology, the Affiliated Weihai Second Municipal Hospital of Qingdao University, 264200, Weihai, Shandong Province, China.*

[#]These authors contributed equally to this work.

[*]Correspondence to: Xiang-ming Mao, Department of Urology, Nanfang Hospital, Southern Medical University. 1838 Guangzhou Avenue North, Tonghe, Baiyun District, Guangzhou, Guangdong Province, China; Phone: +86 13802503635; Fax: +86-20-61641047; Email: mxm631221@126.com. Or, Ya-guang Zou, Department of Stomatology, Nanfang Hospital, Southern Medical University. 1838 Guangzhou Avenue North, Tonghe, Baiyun District, Guangzhou, Guangdong Province, China; Phone: +86-18620050609; Fax: +86-20-61641047; Email : 18620050609@163.com.

Authors ： Hui Qiao: nfyyjxb@163.com; Xiao Tan: 18073125587@163.com; Dao-jun Lv: 15914336377@163.com; Rong, Wei Xing: XRW5096@sina.com; Fang-peng Shu, 374561063@qq.com; Chuan-fan Zhong: 1466778739@qq.com; Chun Li: 120601847@qq.com.

Supplementary data

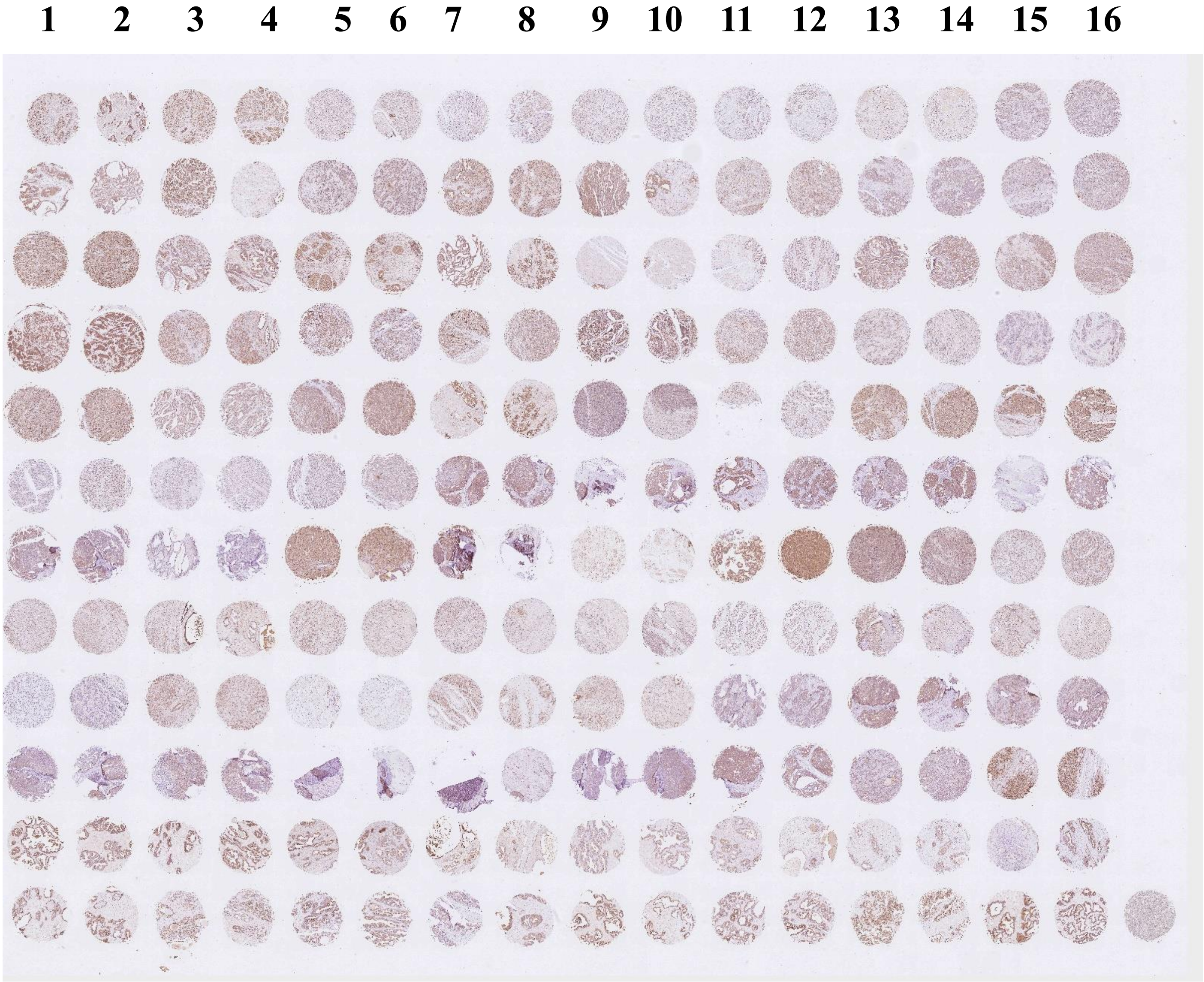

Supplemental Figure 1: The immunohistochemistry image of the whole TMA (scale bar = 1.5 mm).

Supplemental Table 1: The detailed clinic parameters of enrolled patients.
